# Single-dose modified bloodless del Nido cardioplegia for minimally invasive cardiac surgery

**DOI:** 10.3389/fcvm.2025.1448310

**Published:** 2025-02-25

**Authors:** Heemoon Lee, Jihoon Kim, Jae Suk Yoo

**Affiliations:** ^1^ Department of Thoracic and Cardiovascular Surgery, Bucheon Sejong Hospital, Bucheon, Republic of Korea; ^2^ Department of Thoracic and Cardiovascular Surgery, Kangnam Sacred Heart Hospital, Hallym University Medical Center, Hallym University College of Medicine, Seoul, Republic of Korea

**Keywords:** cardioplegia, HTK solution, del Nido cardioplegia solution, minimally invasive cardiac surgery, myocardial protection

## Abstract

**Background:**

Recent studies demonstrated satisfactory results of del Nido cardioplegia in minimally invasive cardiac surgery (MICS). We aimed to evaluate the efficacy of our modified “bloodless” del Nido cardioplegia in MICS compared to the histidine-tryptophan-ketoglutarate (HTK) solution.

**Methods:**

We retrospectively reviewed 471 patients who underwent minimally invasive cardiac surgery (MICS) in our institution between January 2015 and September 2022. Patients were divided into HTK (*n* = 96) and bloodless del Nido (*n* = 375) groups. Using propensity score matching, we matched 72 patients with bloodless del Nido to 72 patients with HTK, based on demographic and operative information.

**Results:**

There were no significant differences in the baseline characteristics and operative data after matching. The early mortality and morbidities did not differ significantly between the two groups. Freedom from overall mortality did not differ significantly during the follow-up period (97.2% in HTK vs. 98.6 in bloodless del Nido at 2 years, *P* = 0.56). The two groups had no difference in postoperative lactate levels at 6 and 24 h. Various statistical methods consistently indicated that bloodless del Nido cardioplegia did not increase risks for overall mortality.

**Conclusions:**

The modified bloodless del Nido cardioplegia showed comparable postoperative outcomes in MICS compared with the HTK solution, suggesting its potential as an alternative option for MICS.

## Introduction

Blood cardioplegia has been acknowledged as a highly effective method for myocardial protection in cardiac surgery (1–3). Despite its benefits, multidose administration requires large solution volumes that can disrupt surgery and increase operation time (4, 5). On the other hand, the histidine-tryptophan-ketoglutarate (HTK) solution provides over two hours of myocardial protection with a single dose yielding favorable clinical outcomes. However, administration of HTK solution may disturb blood homeostasis, resulting in hyponatremia and hemodilution (6–8).

Meanwhile, minimally invasive cardiac surgery (MICS) has become increasingly popular in various cardiac surgeries (9–12). However, myocardial protection during MICS can be particularly challenging due to the limited operative field (13). Administering multiple doses of cardioplegia can be difficult, so “single-dose cardioplegia” is preferred in MICS to avoid repeated adjustments to the surgical field, minimize disruptions to the ongoing procedure, and reduce aortic cross-clamp (ACC) time. As a result, HTK solution has emerged as the gold standard for myocardial protection in MICS and has been shown to produce satisfactory clinical outcomes (13–15).

The del Nido cardioplegia, introduced in the 1990s as a single-dose blood cardioplegic solution initially for congenital and pediatric heart surgeries, has demonstrated a secure window of myocardial protection lasting over 90 min (16, 17). Afterward, the del Nido cardioplegia has also been used in various adult cardiac surgeries and showed favorable outcomes (18–20). Furthermore, recent studies also demonstrated satisfactory results of del Nido cardioplegia in MICS (21, 22). In addition, modifications of del Nido cardioplegia already suggested in several studies and showed favorable results (23–25).

At our institution, adapting the del Nido solution for pediatric cases, we have deliberately excluded blood mixing in pursuing low-volume cardioplegia. Drawing from our accumulating positive institutional experiences, we have seamlessly expanded the use of this “bloodless” del Nido cardioplegia to adults. The objective of the present study is to evaluate the efficacy of our modified bloodless del Nido cardioplegia in MICS compared with the HTK solution.

## Materials and methods

### Study population

We retrospectively reviewed 471 patients who underwent MICS in our institution between January 2015 and September 2022. The median age was 63.0 [53.0–74.0] years, and 230 (48.8%) were female. The median EuroSCORE II value was 1.4 [0.8–3.0]%. Patients were classified into two groups based on the cardioplegic solution: bloodless del Nido (*n* = 375) and HTK (*n* = 96). Performing propensity score matching based on demographic and operative information, we matched 72 patients in the bloodless del Nido group with 72 patients in the HTK group. The study protocol was approved by the Bucheon Sejong Hospital Institutional Review Board, which waived the requirement for patient consent (IRB No. 2023-08-002, approval date September 13, 2023).

### Operative techniques and myocardial protection strategies

All patients underwent cardiac surgery with a minimally invasive approach (right mini-thoracotomy, right anterior thoracotomy, and partial sternotomy). The surgical and cardiopulmonary bypass (CPB) strategies for MICS were described previously (13, 26, 27). Operations were performed under mild hypothermia. Myocardial protection was achieved with cardioplegic arrest. Modified ultrafiltration was performed before decannulation to remove excess fluid and metabolic waste from the blood following cardiac surgery.

### HTK solution

HTK solution was used as the commercially available product (Custodiol®, Köhler Chemie GmbH, Germany). The composition of the HTK solution is described in Table 1. The initial dose of HTK solution was 2000 ml. The cardioplegia was delivered in antegrade fashion into the aortic root for over 7 min with a 4℃ infusion temperature. When ACC time exceeded 120–180 min, an additional dose (half of the initial) was administered.

**Table 1:** Composition of cardioplegic solutions.

HTK		Bloodless del Nido	
Water for injection	1 L	Plasma-Lyte A base solution	1 L
NaCl	0.8766 g	Mannitol 15%	21.7 ml
KCl	0.6710 g	Potassium chloride (2 mEq/ml)	13 ml
MgCl_2_·6H_2_O	0.8132 g	Sodium bicarbonate 8.4%	13 ml
Mannitol	5.4651 g	Magnesium sulfate 50%	4 ml
Histidine	27.9289 g	Lidocaine 1%	13 ml
Tryptophan	0.4085 g	20% Dextrose solution	10 ml
Histidine·HCl·H_2_O	3.7733 g		
CaCl_2_·2H_2_O	0.0022 g		
2-Ketoglutarate-H-K	0.1842 g		

HTK, histidine-tryptophan-ketoglutarate.

### Modified bloodless del Nido cardioplegia

The bloodless del Nido cardioplegic solution contains nearly the same components as the original del Nido solution, with the exception that 15% mannitol is used instead of 20% (Table 1). Unlike the original solution, blood was not mixed during cardioplegia infusion. Instead, 10 ml of 20% Dextrose solution was included as an energy substrate. The cardioplegia was infused antegrade into the aortic root at a temperature of 4℃. The initial dose was 600 ml/m^2^ of body surface area, and if the ACC time exceeded 90 min, an additional dose (half of the initial) was administered every 60 min.

### Endpoints

The primary endpoint of the study was all-cause mortality. The secondary endpoints were postoperative low cardiac output syndrome requiring mechanical circulatory support, postoperative left ventricular ejection fraction (LVEF), postoperative lactate levels, and spontaneous sinus rhythm recovery after ACC release.

### Statistical analysis

Descriptive statistics for the total study population were obtained. Categorical variables were reported as numbers and percentages, while continuous variables were presented as means and standard deviations or median [Q1–Q3]. The inter-group differences were assessed using the *t*-test (or the Mann–Whitney test when the normality assumption was in doubt) and Chi-square test (or Fisher's exact test when the expected cell frequency was <5). Propensity score matching was conducted to reduce differences in baseline characteristics between the groups. The propensity score was obtained by multiple logistic regression, based on the preoperative baseline characteristics (age, sex, New York Heart Association class 3–4, hypertension, diabetes mellitus, dialysis, atrial fibrillation, LVEF ≤40%) and operative data (ACC time, aortic valve replacement, mitral valve replacement, mitral valve repair, tricuspid valve repair, maze operation, aortic surgery). A total of 72 bloodless del Nido group patients were matched to 72 HTK group patients, using nearest-neighbor matching without replacement, and a matching tolerance (caliper) of 0.2. The paired *t*-test and McNemar test were performed after matching. Survival curves were constructed by applying the Kaplan–Meier estimator, and the survival rates were compared between the two groups and subgroups using the log-rank test. The Cox proportional hazards model analysis was utilized to estimate the treatment effect of the two groups on long-term clinical outcomes. The hazard ratios of late clinical outcomes between the two groups were compared using both the original unmatched data and the matched data. Due to the small number of events, we applied Firth's penalized logistic regression. The rates of missingness for data in our models were <1%, and no imputation was performed for missing data. Statistical significance was set at *P* < 0.05. Statistical analysis was carried out using R 4.3.1 (R Foundation for Statistical Computing, Vienna, Austria).

## Results

### Baseline characteristics and operative data

Table 2 summarizes the baseline characteristics of the patients. Before matching, patients in the bloodless del Nido group had a higher prevalence of New York Heart Association class 3–4 (HTK vs. bloodless del Nido; 13.5% vs. 33.1%, *P* < 0.01), atrial fibrillation (8.3% vs. 20.8%, *P* = 0.01), previous open heart surgery (4.2% vs. 12.5%, *P* = 0.03) and higher EuroSCORE [1.1 [0.8–1.9]% vs. 1.6 [0.8–3.4]%, *P* = 0.01]. However, no differences in demographic data were observed between the groups after propensity score matching.

**Table 2:** Baseline characteristics.

Variables	Before matching				After matching			
	HTK (*n* = 96)	Bloodless del Nido (*n* = 375)	*P* value	SMD	HTK (*n* = 72)	Bloodless del Nido (*n* = 72)	*P* value	SMD
Age, years	66.0 [59.0–73.0]	61.0 [51.5–75.0]	0.18	0.211	66.0 [59.0–74.0]	66.0 [59.0–74.0]	0.39	0.150
Female, *n* (%)	43 (44.8)	187 (49.0)	0.44	0.102	35 (48.6)	39 (54.2)	0.64	0.111
NYHA class 3–4, *n* (%)	13 (13.5)	124 (33.1)	<0.01	0.475	13 (18.1)	6 (8.3)	0.12	0.290
Hypertension, *n* (%)	51 (53.1)	180 (48.0)	0.43	0.103	38 (52.8)	33 (45.8)	0.51	0.139
Diabetes mellitus, *n* (%)	28 (29.2)	73 (19.5)	0.05	0.228	19 (26.4)	16 (22.2)	0.68	0.097
Stroke, *n* (%)	8 (8.3)	27 (7.2)	0.87	0.042	6 (8.3)	4 (5.6)	0.75	0.109
Atrial fibrillation, *n* (%)	8 (8.3)	78 (20.8)	0.01	0.359	8 (11.1)	6 (8.3)	0.79	0.094
COPD, *n* (%)	3 (3.1)	15 (4.0)	0.92	0.047	2 (2.8)	4 (5.6)	0.68	0.139
Dialysis, *n* (%)	3 (3.1)	5 (1.3)	0.44	0.122	2 (2.8)	2 (2.8)	>0.99	<0.001
EuroSCORE, %	1.1 [0.8–1.9]	1.6 [0.8–3.4]	0.01	0.264	1.1 [0.7–2.0]	1.3 [0.8–2.1]	0.39	0.156
Previous OHS, *n* (%)	4 (4.2)	47 (12.5)	0.03	0.306	4 (5.6)	4 (5.6)	>0.99	<0.001
LVEF ≤40%	5 (5.2)	9 (2.4)	0.27	0.147	5 (6.9)	2 (2.8)	0.45	0.195

HTK, histidine-tryptophan-ketoglutarate; SMD, standardized mean difference; NYHA, New York heart association; COPD, chronic obstructive pulmonary disease; OHS, open heart surgery; LVEF, left ventricular ejection fraction.

Operative data are described in Table 3. CPB time, ACC time, and operative procedures did not differ between the two groups after matching.

**Table 3:** Operative data.

Variables	Before matching				After matching			
	HTK (*n* = 96)	Bloodless del Nido (*n* = 375)	*P* value	SMD	HTK (*n* = 72)	Bloodless del Nido (*n* = 72)	*P* value	SMD
CPB time, min	150.0 [117.0–176.2]	134.0 [102.0–175.0]	<0.01	0.181	134.5 [113.5–157.0]	131.0 [110.0–218.0]	0.85	0.221
ACC time, min	115.0 [90.5–138.3]	89.0 [64.0–118.5]	<0.01	0.617	100.0 [85.0–126.0]	96.5 [79.5–130.0]	0.37	0.052
No. of cardioplegia infusions	1.0 [1.0–1.0]	1.0 [1.0–2.0]	0.09	0.255	1.0 [1.0–1.0]	1.0 [1.0–2.0]	0.02	0.446
Second dose of cardioplegia, *n* (%)	17 (17.7)	98 (25.6)	0.138	0.192	13 (18.1)	24 (33.3)	0.03	0.355
Third dose of cardioplegia, *n* (%)	0 (0.0)	13 (3.5)	0.133	0.268	0 (0.0)	7 (9.7)	0.02	0.464
Spontaneous sinus rhythm recovery after ACC release, *n* (%)	48 (50.0)	343 (91.5)	<0.01	1.024	35 (48.6)	63 (87.5)	<0.01	0.918
Operative procedures								
AVR, *n* (%)	73 (76.0)	157 (41.9)	<0.01	0.741	55 (76.4)	52 (72.2)	0.65	0.095
MVR, *n* (%)	8 (8.3)	164 (43.7)	<0.01	0.882	8 (11.1)	11 (15.3)	0.55	0.123
MV repair, *n* (%)	2 (2.1)	117 (31.2)	<0.01	0.849	2 (2.8)	5 (6.9)	0.37	0.195
TV repair, *n* (%)	10 (10.4)	66 (17.6)	0.12	0.208	10 (13.9)	8 (11.1)	0.79	0.084
Maze operation, *n* (%)	4 (4.2)	37 (9.9)	0.12	0.225	4 (5.6)	3 (4.2)	>0.99	0.065
Aortic surgery, *n* (%)	9 (9.4)	3 (0.8)	<0.01	0.398	1 (1.4)	2 (2.8)	>0.99	0.097
ASD closure, *n* (%)	0 (0.0)	21 (5.6)	0.04	0.344	0 (0.0)	2 (2.8)	0.48	0.239

HTK, histidine-tryptophan-ketoglutarate; SMD, standardized mean difference; CPB, cardiopulmonary bypass; ACC, aortic cross-clamp; AVR, aortic valve replacement; MVR, mitral valve replacement; MV, mitral valve; TV, tricuspid valve; ASD, atrial septal defect.

The number of cardioplegia infusions was significantly higher in the bloodless del Nido group (*P* = 0.02). The requirement for second and third doses of cardioplegia was also higher in the bloodless del Nido group (*P* = 0.03 and 0.02, respectively). The spontaneous sinus rhythm recovery rate after ACC release was significantly higher in the bloodless del Nido group (*P* < 0.01).

### Outcomes

**Figure 1:**
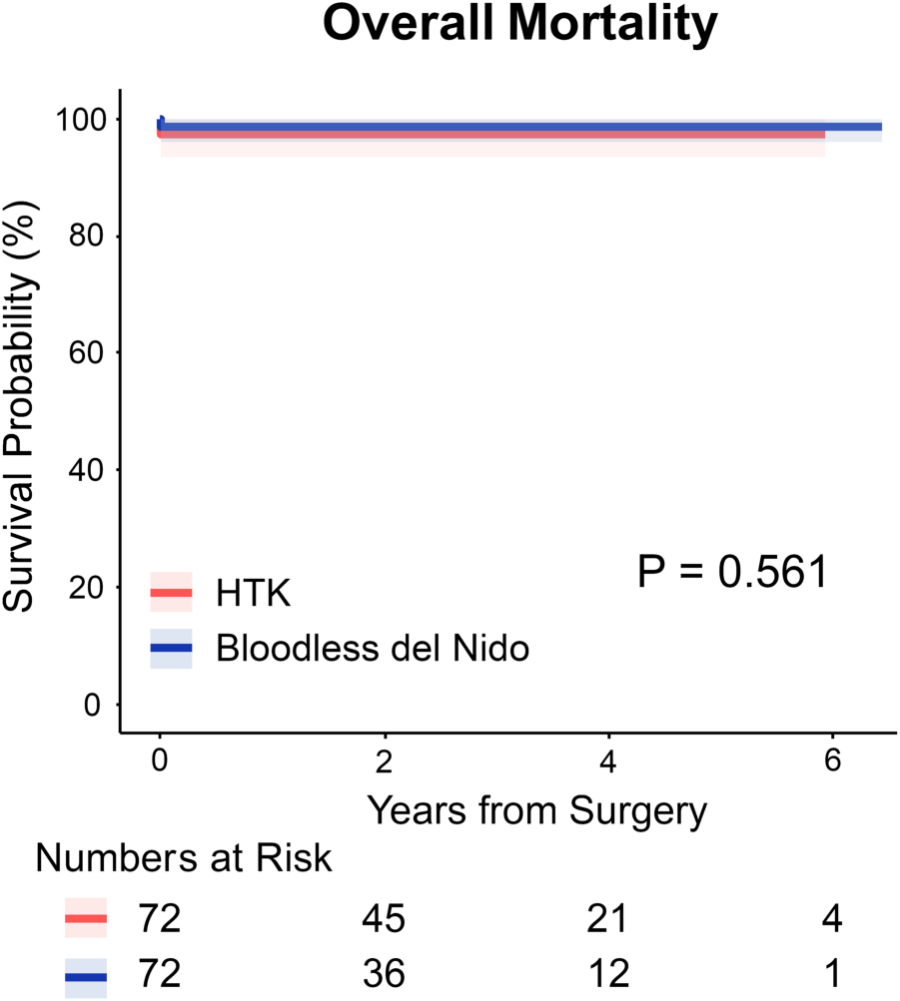
Kaplan–meier survival curves for overall survival in HTK and bloodless del Nido group. HTK, histidine-tryptophan-ketoglutarate.

Figure 1 shows Kaplan–Meier survival curves for overall mortality. Freedom from overall mortality did not differ significantly between the two groups (97.2% in HTK vs. 98.6% in bloodless del Nido at 2 years, *P* = 0.56).

**Figure 2:**
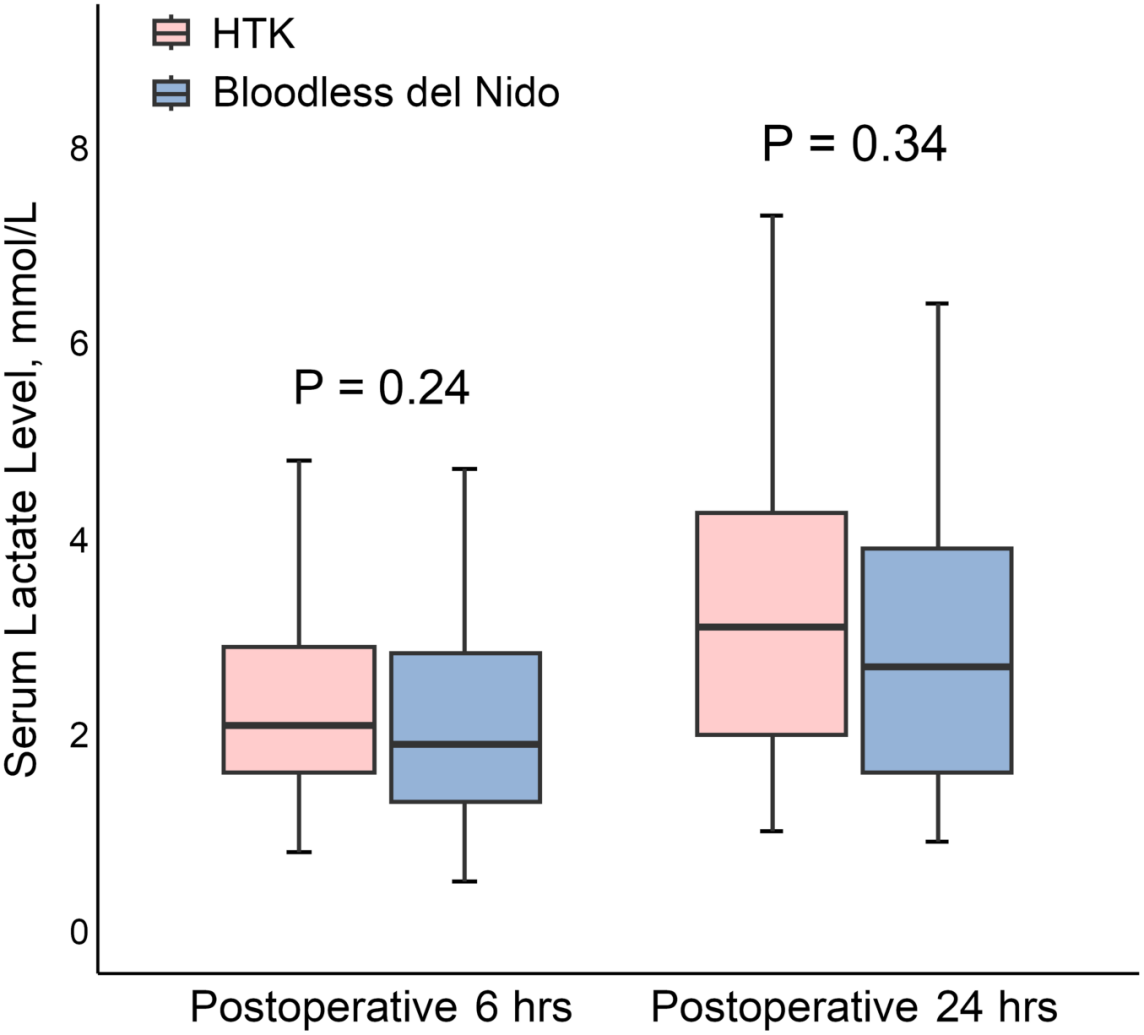
Postoperative lactate levels at 6 and 24 h after surgery. HTK, histidine-tryptophan-ketoglutarate.

Postoperative lactate levels at 6 and 24 h are shown in Figure 2. The HTK group exhibited higher lactate levels than the bloodless del Nido group at both time points; however, the differences were not statistically significant (HTK vs. bloodless del Nido at 6 h: 2.1 [1.6–3.2] vs. 1.9 [1.3–2.9] mmol/L, *P* = 0.24; at 24 h: 3.4 [2.0–4.4] vs. 2.8 [1.6–4.2] mmol/L, *P* = 0.34).

**Figure 3:**
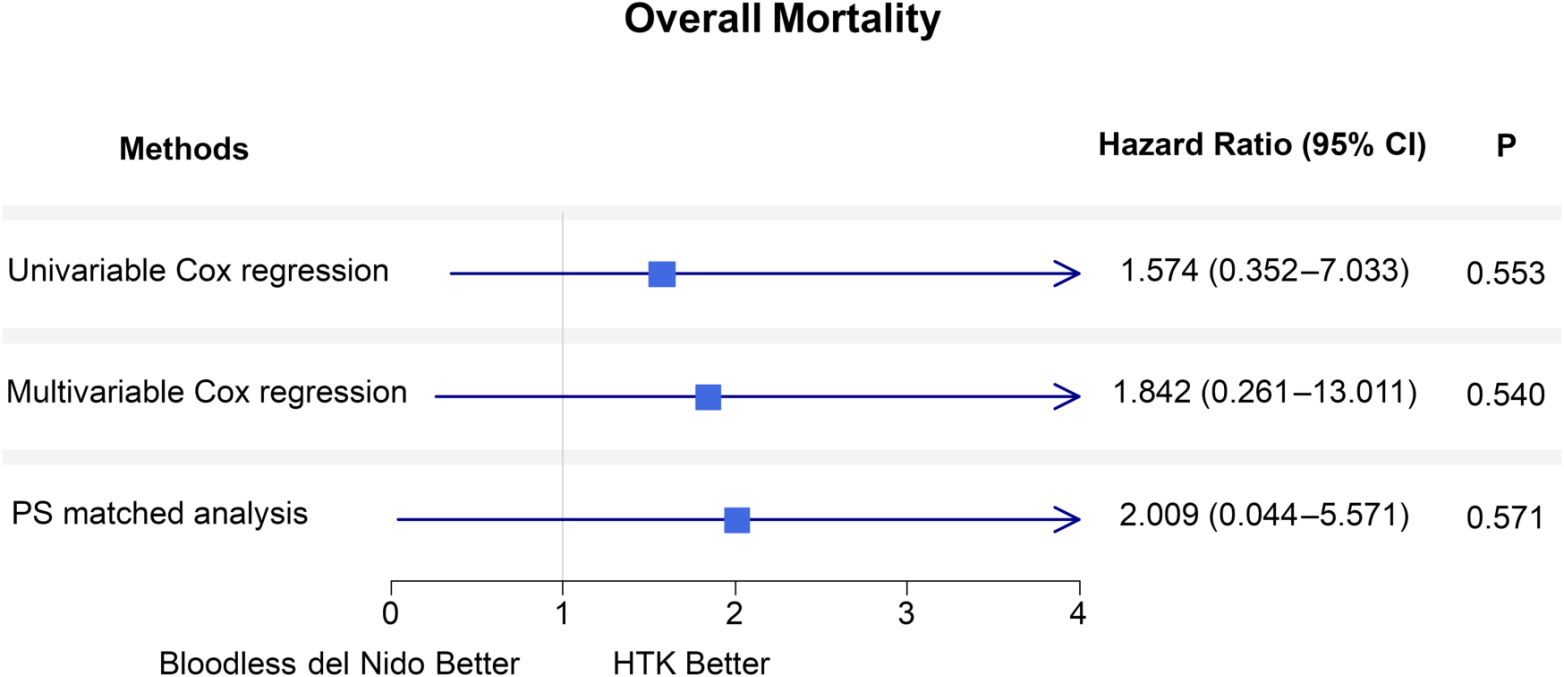
Forest plot of hazard ratios for overall mortality comparing the HTK and bloodless del nido groups during the follow-up period using the Cox proportional hazard models. Analyses are performed with univariable, multivariable Cox regression, and propensity score-matched analysis. CI, confidence interval; PS, propensity score; HTK, histidine-tryptophan-ketoglutarate.

Figure 3 summarizes the hazard ratios for overall mortality between the two groups. Various statistical methods consistently indicated that bloodless del Nido cardioplegia did not increase risks for overall mortality. The results of univariable and multivariable Cox regression analysis was demonstrated in Supplementary Table S1. Firth's penalized logistic regression yielded consistent results (Supplementary Table S2).

The two groups (HTK vs. bloodless del Nido) did not differ significantly in early mortality (2.8% vs. 1.4%, *P* > 0.99), low cardiac output syndrome requiring mechanical circulatory support (2.8% vs. 2.8%, *P* > 0.99), postoperative LVEF [58.0 [50.0–60.0] vs. 58.0 [52.0–60.0]%, *P* = 0.79], and other early morbidities.

Although the amount of intraoperative red blood cell (RBC) transfusion was significantly greater in the HTK group, the amount of postoperative RBC transfusion did not differ between the two groups. The duration of mechanical ventilation was longer in the HTK group. The initial postoperative serum Na^+^ level was significantly lower in the HTK group. There were no significant differences in the lengths of stay in the intensive care unit between the two groups (Table 4).

**Table 4:** Early outcomes.

Variables	Before matching				After matching			
	HTK (*n* = 96)	Bloodless del Nido (*n* = 375)	*P* value	SMD	HTK (*n* = 72)	Bloodless del Nido (*n* = 72)	*P* value	SMD
Postoperative LVEF, %	58.0 [52.5–60.0]	58.0 [51.0–60.0]	0.40	0.049	58.0 [50.0–60.0]	58.0 [52.0–60.0]	0.79	0.073
Bleeding reoperation, *n* (%)	9 (6.2)	12 (3.2)	0.28	0.144	5 (6.9)	1 (1.4)	0.22	0.281
AKI, *n* (%)	7 (7.3)	9 (2.4)	0.04	0.229	5 (6.9)	4 (5.6)	>0.99	0.057
LCOS, *n* (%)	4 (4.2)	11 (2.9)	0.77	0.067	2 (2.8)	2 (2.8)	>0.99	<0.001
Stroke, *n* (%)	2 (2.1)	4 (1.1)	0.78	0.082	2 (2.8)	0 (0.0)	0.48	0.239
Pneumonia, *n* (%)	1 (1.0)	5 (1.3)	>0.99	0.027	1 (1.4)	1 (1.4)	>0.99	<0.001
Mortality, *n* (%)	2 (2.1)	12 (3.2)	0.81	0.065	2 (2.8)	1 (1.4)	>0.99	0.097
Intraoperative RBC transfusion, pack	2.0 [0–3.0]	0 [0–2.0]	<0.01	0.377	2.0 [0–3.0]	0 [0–2.0]	<0.01	0.474
Postoperative RBC transfusion, pack	0 [0–3.0]	0 [0–0]	0.01	0.183	0 [0–2.0]	0 [0–0]	0.05	0.238
Initial postoperative serum Na^+^ concentration, mmol/L	138.0 [136.0–140.0]	141.0 [139.0–143.0]	<0.01	0.831	137.7 ± 3.1	140.9 ± 3.3	<0.01	0.992
Duration of mechanical ventilation, min	375.0 [305.0–532.5]	360.0 [270.0–502.5]	0.21	0.175	380.0 [307.5–540.5]	347.5 [260.0–467.5]	0.07	0.352
ICU stay, days	1.0 [1.0–2.0]	1.0 [1.0–1.0]	0.04	0.035	1.0 [1.0–2.0]	1.0 [1.0–1.5]	0.59	0.148
Hospital stay, days	8.0 [7.0–13.0]	7.0 [6.0–10.0]	<0.01	0.176	8.0 [7.0–13.0]	7.0 [6.0–10.5]	0.02	0.168

HTK, histidine-tryptophan-ketoglutarate; SMD, standardized mean difference; LVEF, left ventricular ejection fraction; AKI, acute kidney injury; LCOS, low cardiac output syndrome; RBC, red blood cell; ICU, intensive care unit.

## Discussion

Our study showed that modified bloodless del Nido cardioplegia in MICS had acceptable outcomes compared to the HTK solution. The two groups had no significant difference in early mortality, overall mortality, or postoperative lactate levels.

Adequate myocardial protection in MICS can be challenging due to limited working space with small incisions and longer ACC times. Administering multiple doses of cardioplegia in these situations can be difficult and risky, potentially prolonging ACC times. Thus, single-dose cardioplegia is often the preferred choice (13).

The HTK solution, a single-dose cardioplegia, offers extended myocardial protection for up to 180 min and is widely utilized in MICS. Several studies have shown favorable results associated with the HTK solution for MICS. Savini et al. described 49 patients who underwent minimally invasive mitral valve surgery (MIMVS) with HTK solution, showing no significant rise in cardiac enzymes and no occurrences of in-hospital mortality or low cardiac output syndromes (14). Matzelle et al. studied 100 patients who underwent MIMVS compared to 113 sternotomy cases. In MIMVS, at 6 h post-surgery, only 12% required continuous inotropic support, and 2 patients needed intra-aortic balloon pump assistance. The median peak troponin-I level within the first 24 h, excluding cryoablation cases, was 5.1 µg/L (range: 0.8–40 µg/L), and there were no instances of early or one-year mortality (15).

On the other hand, del Nido cardioplegia was initially developed as a single-dose cardioplegic solution for congenital and pediatric cardiac procedures (16, 17), and its usage in adult cardiac surgeries has been growing. Moreover, there have been reports of favorable outcomes using the del Nido cardioplegia in MICS. For instance, Gerber et al. reported on matched patients who underwent MIMVS, with 38 receiving the del Nido solution and 38 receiving HTK. The study demonstrated similar outcomes for the del Nido and HTK solutions, including no instances of mortality or myocardial infarction, comparable levels of cardiac enzymes, similar durations of intensive care unit stay, ventilation time, perioperative red blood cell use, and hemoglobin levels (21). In another study, Malvindi et al. described matched 110 patients who underwent MIMVS (55 HTK vs. 55 del Nido). There was no in-hospital mortality. Postoperative cardiac enzyme levels did not differ significantly between the two groups (22). Our study also demonstrated similar outcomes in the bloodless del Nido group compared with the HTK group in MICS. Postoperative lactate level was not significantly different between the two groups.

Earlier research indicated that the del Nido and HTK solutions provide similar myocardial protection. Nonetheless, the del Nido cardioplegia offers distinct advantages over the HTK solution, such as a reduced cardioplegic solution volume, mitigating hemodilution, an increased likelihood of spontaneous rhythm recovery, and cost-effectiveness (28). Our study also demonstrated that the spontaneous sinus rhythm recovery rate after ACC release was significantly higher in the bloodless del Nido group. The initial postoperative serum Na^+^ concentration was significantly lower in the HTK group, suggesting hemodilution (*P* < 0.01). However, the absolute serum Na^+^ value in the HTK group was 137.7 ± 3.1 mmol/L, which may not have clinical significance. Additionally, in our institution, modified ultrafiltration is routinely performed during adult cardiac surgery, which may also affect electrolyte levels.

The length of myocardial protection with a single-dose administration is a key consideration in cardioplegia. Guim et al. suggested that the duration of myocardial protection with del Nido cardioplegia lasts up to 120 min (29). In the study by Ong et al., the authors recommended additional administration 60–90 min after the initial dose (30). At our institution, a second dose of bloodless del Nido cardioplegia was administered 90 min after the initial dose, which was shorter than the administration interval for HTK solution (120–180 min). Although the need for the second or third dose was significantly higher in the bloodless del Nido group in our study, CPB and ACC times were not significantly different between the two groups. Therefore, the administration of a second or third dose does not appear to disrupt the procedures. Additionally, clinical impact may not be significant, as the incidence of low cardiac output syndrome and postoperative lactate levels did not differ significantly between the two groups.

The original del Nido cardioplegia solution includes blood, which provides several theoretical advantages, such as buffering properties, improved coronary perfusion, enhanced oxygen delivery, and reduced ischemic stress and reperfusion injury (31). However, several investigations have proposed that crystalloid cardioplegia, such as the HTK solution, can achieve comparable outcomes to blood cardioplegia (32, 33). In our institution, a modified bloodless cardioplegia has been employed since the early 2000 s for congenital heart surgery. Following its successful outcomes in congenital heart surgery (34), it was integrated into adult cardiac surgery in 2014. Our study also revealed that the modified bloodless del Nido cardioplegia in MICS produced favorable results compared to the HTK solution. Consequently, we posit that the inclusion of blood components may not be imperative for del Nido cardioplegia.

The modified bloodless del Nido cardioplegia also offers specific advantages in particular scenarios. For instance, it could be beneficial in the case of small neonates, infants, or even small adults, where incorporating the patient's blood into the cardioplegia can pose challenges. It is a valuable option in situations with a shortage of blood products or when equipment for mixing blood with cardioplegia is unavailable. Moreover, the cost of the del Nido solution is significantly lower than that of the HTK solution (1 L del Nido: $8 vs. 1 L HTK: $105), rendering it a more financially accessible choice.

Our study has several limitations. First, it is a retrospective, non-randomized study conducted at a single institution, where the selection of cardioplegia was based on the surgeon's preference and clinical conditions. Although PS matching was performed to adjust for potential biases in patient selection, the impact of unaccounted confounding factors on our results cannot be ruled out. Additionally, the relatively small sample size (72 patients per group after PS matching) and the follow-up duration of 28.9 [14.5–49.9] months may limit the ability to detect clinically meaningful differences and assess long-term outcomes. Second, data on cardiac markers such as troponin I and creatine kinase-muscle/brain were unavailable due to the absence of routine testing at our institution, except in coronary artery bypass grafting cases. Moreover, our study did not include other parameters that may indirectly suggest myocardial injury, such as the incidence of postoperative ventricular fibrillation, atrial fibrillation, inotropic support, and the dose of inotropic agents. Postoperative lactate levels were used as an indirect indicator of postoperative low cardiac output state instead of these markers; however, the precise effects of cardioplegias on myocardial function remain unclear. Finally, although bloodless del Nido cardioplegia is less costly than the HTK solution, an analysis of resource utilization and cost-effectiveness was not performed in this study. Taken together, multi-center randomized controlled trials with larger patient populations and longer follow-up durations are needed to substantiate our findings.

## Conclusion

The modified bloodless del Nido cardioplegia demonstrated comparable postoperative outcomes in MICS compared to the HTK solution. It is a potential alternative for MICS procedures and offers particular advantages, especially in smaller patients or under resource-constrained conditions. However, larger multi-center randomized controlled trials are needed to further validate these initial findings.

## Data Availability

The data that support the findings of this study are available from the corresponding author upon reasonable request.

## References

[1] Braathen B Tonnessen T. Cold blood cardioplegia reduces the increase in cardiac enzyme levels compared with cold crystalloid cardioplegia in patients undergoing aortic valve replacement for isolated aortic stenosis. J Thorac Cardiovasc Surg. (2010) 139(4):874–80. 10.1016/j.jtcvs.2009.05.036 19660338

[2] Guru V Omura J Alghamdi AA Weisel R Fremes SE. Is blood superior to crystalloid cardioplegia? A meta-analysis of randomized clinical trials. Circulation. (2006) 114(1):I331–8. 10.1161/CIRCULATIONAHA.105.001644 16820596

[3] Dar MI. Cold crystalloid versus warm blood cardioplegia for coronary artery bypass surgery. Ann Thorac Cardiovasc Surg. (2005) 11(6):382–5. 16401986

[4] Viana FF Shi WY Hayward PA Larobina ME Liskaser F Matalanis G. Custodiol versus blood cardioplegia in complex cardiac operations: an Australian experience. Eur J Cardiothorac Surg. (2013) 43(3):526–31. 10.1093/ejcts/ezs319 22665382

[5] Gambardella I Gaudino MFL Antoniou GA Rahouma M Worku B Tranbaugh RF Single- versus multidose cardioplegia in adult cardiac surgery patients: a meta-analysis. J Thorac Cardiovasc Surg. (2020) 160(5):1195–202.e12. 10.1016/j.jtcvs.2019.07.109 31590948

[6] Lindner G Zapletal B Schwarz C Wisser W Hiesmayr M Lassnigg A. Acute hyponatremia after cardioplegia by histidine-tryptophane-ketoglutarate–a retrospective study. J Cardiothorac Surg. (2012) 7:52. 10.1186/1749-8090-7-52 22681759 PMC3430602

[7] Li XW Lin YZ Lin H Huang JB Tang XM Long XM Histidine-tryptophan-ketoglutarate solution decreases mortality and morbidity in high-risk patients with severe pulmonary arterial hypertension associated with complex congenital heart disease: an 11-year experience from a single institution. Braz J Med Biol Res. (2016) 49(6):e5208. 10.1590/1414-431x20165208 27191607 PMC4869826

[8] Stammers AH Tesdahl EA Mongero LB Stasko AJ Weinstein S. Does the type of cardioplegic technique influence hemodilution and transfusion requirements in adult patients undergoing cardiac surgery? J Extra Corpor Technol. (2017) 49(4):231–40. 10.1051/ject/201749231 29302113 PMC5737423

[9] Paparella D Fattouch K Moscarelli M Santarpino G Nasso G Guida P Current trends in mitral valve surgery: a multicenter national comparison between full-sternotomy and minimally-invasive approach. Int J Cardiol. (2020) 306:147–51. 10.1016/j.ijcard.2019.11.137 31810816

[10] Malaisrie SC Barnhart GR Farivar RS Mehall J Hummel B Rodriguez E Current era minimally invasive aortic valve replacement: techniques and practice. J Thorac Cardiovasc Surg. (2014) 147(1):6–14. 10.1016/j.jtcvs.2013.08.086 24183904

[11] Shin C Ju MH Lee CH Lim MH Je HG. Surgical outcomes of cardiac myxoma resection through right mini-thoracotomy. J Chest Surg. (2023) 56(1):42–8. 10.5090/jcs.22.094 36517950 PMC9845859

[12] Choi JW Kim JB Jung YJ Hwang HY Kim KH Yoo JS Trends in heart valve surgery in Korea: a report from the heart valve surgery registry database. J Chest Surg. (2022) 55(5):388–96. 10.5090/jcs.22.016 35999692 PMC9579849

[13] Garbade J Davierwala P Seeburger J Pfannmueller B Misfeld M Borger MA Myocardial protection during minimally invasive mitral valve surgery: strategies and cardioplegic solutions. Ann Cardiothorac Surg. (2013) 2(6):803–8. 10.3978/j.issn.2225-319X.2013.09.04 24349985 PMC3856986

[14] Savini C Murana G Di Eusanio M Suarez SM Jafrancesco G Castrovinci S Safety of single-dose histidine-tryptophan-ketoglutarate cardioplegia during minimally invasive mitral valve surgery. Innovations. (2014) 9(6):416–20. 10.1177/155698451400900604 25251549

[15] Matzelle SJ Murphy MJ Weightman WM Gibbs NM Edelman JJ Passage J. Minimally invasive mitral valve surgery using single dose antegrade Custodiol cardioplegia. Heart Lung Circ. (2014) 23(9):863–8. 10.1016/j.hlc.2014.03.018 24767979

[16] Matte GS del Nido PJ. History and use of del Nido cardioplegia solution at Boston Children’s hospital. J Extra Corpor Technol. (2012) 44(3):98–103. 10.1051/ject/201244098 23198389 PMC4557532

[17] Charette K Gerrah R Quaegebeur J Chen J Riley D Mongero L Single dose myocardial protection technique utilizing del Nido cardioplegia solution during congenital heart surgery procedures. Perfusion. (2012) 27(2):98–103. 10.1177/0267659111424788 22005886

[18] Ad N Holmes SD Massimiano PS Rongione AJ Fornaresio LM Fitzgerald D. The use of del Nido cardioplegia in adult cardiac surgery: a prospective randomized trial. J Thorac Cardiovasc Surg. (2018) 155(3):1011–8. 10.1016/j.jtcvs.2017.09.146 29246552 PMC5929134

[19] Yerebakan H Sorabella RA Najjar M Castillero E Mongero L Beck J Del Nido Cardioplegia can be safely administered in high-risk coronary artery bypass grafting surgery after acute myocardial infarction: a propensity matched comparison. J Cardiothorac Surg. (2014) 9:141. 10.1186/s13019-014-0141-5 25359427 PMC4220058

[20] Kim JS Jeong JH Moon SJ Ahn H Hwang HY. Sufficient myocardial protection of del Nido cardioplegia regardless of ventricular mass and myocardial ischemic time in adult cardiac surgical patients. J Thorac Dis. (2016) 8(8):2004–10. 10.21037/jtd.2016.06.66 27621853 PMC4999689

[21] Gerber W Sanetra K Gerber AD Jankowska-Sanetra J Kuczera M Bialek K One-shot cardioplegia for minimally invasive mitral valve repair-a comparison of del Nido and Bretschneider Histidine-Tryptophan-Ketoglutarate solutions. Perfusion. (2023) 38(4):763–70. 10.1177/02676591221080653 35320027

[22] Malvindi PG Bifulco O Berretta P Silvano R Alfonsi J Cefarelli M del Nido and Histidine-Tryptophan-Ketoglutarate cardioplegia in minimally invasive mitral valve surgery: a propensity-Match study. Perfusion. (2023) 39(4):823–32. 10.1177/02676591231161920 36881663

[23] Taka H Douguchi T Miyamoto A Shimizu K Kimura S Iwasaki T Modified del Nido cardioplegia is associated with low incidence of low main strong ion difference and hyperchloremia in pediatric patients after cardiac surgery. J Anesth. (2024) 38(2):244–53. 10.1007/s00540-023-03306-0 38358399

[24] Sithiamnuai P Tocharoenchok T. Modified del Nido versus blood cardioplegia in congenital cardiac surgery. Asian Cardiovasc Thorac Ann. (2022) 30(5):555–60. 10.1177/02184923211048332 34553609

[25] Brown S Nassar K Razzouk J Kashyap AK Won M Shehadeh T Outcomes of coronary artery bypass surgery using modified del nido cardioplegia in patients with poor ventricular function. J Cardiothorac Surg. (2023) 18(1):346. 10.1186/s13019-023-02466-0 38031138 PMC10685478

[26] Lee H Kim J Jung JH Yoo JS. Midterm outcomes of isolated tricuspid valve surgery with a mini-thoracotomy and beating heart strategy. J Thorac Dis. (2023) 15(6):3126–32. 10.21037/jtd-22-1868 37426123 PMC10323552

[27] Bakhtiary F Salamate S Amer M Sirat S Bayram A Doss M Comparison of right anterior mini-thoracotomy versus partial upper sternotomy in aortic valve replacement. Adv Ther. (2022) 39(9):4266–84. 10.1007/s12325-022-02263-6 35906515 PMC9402480

[28] Duan L Hu GH Wang E Zhang CL Huang LJ Duan YY. Del Nido versus HTK cardioplegia for myocardial protection during adult complex valve surgery: a retrospective study. BMC Cardiovasc Disord. (2021) 21(1):604. 10.1186/s12872-021-02411-w 34922443 PMC8683821

[29] Guim GS Wah Hoon CG Lim CA Chay-Nancy HS Li Ler AA Lim QX Use of del nido cardioplegia for adult heart surgery: how long is not too long? J Extra Corpor Technol. (2020) 52(4):272–8. 10.1051/ject/202052272 33343029 PMC7728503

[30] Ong GS Guim GS Lim QX Chay-Nancy HS Jaafar NB Lim CA Alternative technique of long acting cardioplegia delivery results in less hemodilution. Perfusion. (2021) 36(4):365–73. 10.1177/0267659120946727 32777980

[31] Bhakri KP Mulholland J Punjabi PP. Understanding innovations in the evolving practice of blood and crystalloid cardioplegia. Perfusion. (2014) 29(6):505–10. 10.1177/0267659114524977 24609840

[32] Albadrani M. Histidine-tryptophan-ketoglutarate solution versus multidose cardioplegia for myocardial protection in cardiac surgeries: a systematic review and meta-analysis. J Cardiothorac Surg. (2022) 17(1):133. 10.1186/s13019-022-01891-x 35642063 PMC9158226

[33] Lerman DA Otero-Losada M Ume K Salgado PA Prasad S Lim K Is cold blood cardioplegia absolutely superior to cold crystalloid cardioplegia in aortic valve surgery? J Cardiovasc Surg. (2018) 59(1):115–20. 10.23736/S0021-9509.17.09979-7 28548476 PMC7099971

[34] Kim ER Lee CH Kim WH Lim JH Kim YJ Min J Primary versus staged repair in neonates with pulmonary atresia and ventricular septal defect. Ann Thorac Surg. (2021) 112(3):825–30. 10.1016/j.athoracsur.2020.06.098 32896547

