# Corrigendum: Single-dose modified bloodless del Nido cardioplegia for minimally invasive cardiac surgery

**DOI:** 10.3389/fcvm.2025.1603181

**Published:** 2025-05-09

**Authors:** Heemoon Lee, Jihoon Kim, Jae Suk Yoo

**Affiliations:** ^1^ Department of Thoracic and Cardiovascular Surgery, Bucheon Sejong Hospital, Bucheon, Republic of Korea; ^2^ Department of Thoracic and Cardiovascular Surgery, Kangnam Sacred Heart Hospital, Hallym University Medical Center, Hallym University College of Medicine, Seoul, Republic of Korea

**Keywords:** cardioplegia, HTK solution, del Nido cardioplegia solution, minimally invasive cardiac surgery, myocardial protection

A Corrigendum on Single-dose modified bloodless del Nido cardioplegia for minimally invasive cardiac surgery By Lee H, Kim J and Yoo JS (2025). *Front Cardiovasc Med.* 12:1448310. doi: 10.3389/fcvm.2025.1448310

In the published article, there was an error in Table 1 as published. The volume of Mannitol 15% in the Bloodless del Nido cardioplegia is incorrectly listed as 16.3 ml. The correct volume should be 21.7 ml. The corrected Table 1 and its caption appear below.

**Table 1:** Composition of cardioplegic solutions.

HTK		Bloodless del Nido	
Water for injection	1 L	Plasma-Lyte A base solution	1 L
NaCl	0.8766 g	Mannitol 15%	21.7 ml
KCl	0.6710 g	Potassium chloride (2 mEq/ml)	13 ml
MgCl_2_.6H_2_O	0.8132 g	Sodium bicarbonate 8.4%	13 ml
Mannitol	5.4651 g	Magnesium sulfate 50%	4 ml
Histidine	27.9289 g	Lidocaine 1%	13 ml
Tryptophan	0.4085 g	20% dextrose solution	10 ml
Histidine.HCl.H_2_O	3.7733 g		
CaCl_2_.2H_2_O	0.0022 g		
2-Ketoglutarate-H-K	0.1842 g		

HTK, histidine-tryptophan-ketoglutarate.

The authors apologize for this error and state that this does not change the scientific conclusions of the article in any way. The original article has been updated.

