# Supplementary material for: Single-dose modified bloodless del Nido cardioplegia for minimally invasive cardiac surgery
Source: Front Cardiovasc Med. 2025 Feb 25;12:1448310. doi: 10.3389/fcvm.2025.1448310 (PMC11893588; doi:10.3389/fcvm.2025.1448310)
Supplement: Supplementary file 1 [file Table1.docx]

**Table S1**. Cox regression analysis for overall mortality

| Variable | P value  (univariable) | P value  (multivariable) | HR | 95% CI |
| --- | --- | --- | --- | --- |
| Group | 0.553 | 0.540 | 1.842 | 0.261–13.011 |
| NYHA class 3–4 | 0.001 |  |  |  |
| Stroke | 0.006 |  |  |  |
| Atrial fibrillation | <0.001 |  |  |  |
| Dialysis | 0.135 |  |  |  |
| Previous OHS | <0.001 |  |  |  |
| EuroSCORE | <0.001 |  |  |  |
| CPB time | <0.001 |  |  |  |
| ACC time | <0.001 |  |  |  |

*HR*, hazard ratio; *CI*, confidence interval; *NYHA*, New York Heart Association; *OHS*, open heart surgery; *CPB*, cardiopulmonary bypass; *ACC*, aortic cross-clamp.

**Table S2.** Firth’s penalized logistic regression analysis for overall mortality

| Method | OR | 95% CI | P value |
| --- | --- | --- | --- |
| Univariable | 0.130 | 0.381–6.734 | 0.701 |
| Multivariable | 0.137 | 0.244–13.965 | 0.741 |
| Propensity matched analysis | 0.592 | 0.053–4.559 | 0.610 |

*OR*, odds ratio; *CI*, confidence interval.
